# Latent pH-responsive ratiometric fluorescent cluster based on self-assembled photoactivated SNARF derivatives

**DOI:** 10.1080/14686996.2016.1204888

**Published:** 2016-08-02

**Authors:** Eiji Nakata, Yoshihiro Yukimachi, Yoshihiro Uto, Hitoshi Hori, Takashi Morii

**Affiliations:** ^a^ Institute of Advanced Energy, Kyoto University, Uji, Kyoto, Japan; ^b^ Department of Life System, Institute of Technology and Science, Graduate School, The University of Tokushima, Tokushima, Japan

**Keywords:** Self-assembly, fluorescent material, ratiometry, pH indicator, photoactivation, 30 Bio-inspired and biomedical materials, 101 Self-assembly / Self-organized materials, 208 Sensors and actuators

## Abstract

We have developed a self-assembled fluorescent cluster comprising a seminaphthorhodafluor (SNARF) derivative protected by a photoremovable *o*-nitrobenzyl group. Prior to UV irradiation, a colorless and nonfluorescent cluster was spontaneously assembled in aqueous solution. After UV irradiation, the self-assembled cluster remained intact and showed a large enhancement in pH-responsive fluorescence. The unique pH responsive fluorescent cluster could be used as a dual-emissive ratiometric fluorescent pH probe not only in the test tube but also in HeLa cell cultures.

## Graphical abstract

**Figure:**
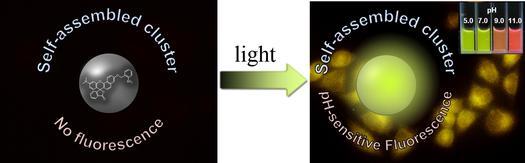

## 1. Introduction

Fluorescence imaging is one of the most powerful techniques for the real-time, noninvasive monitoring of biomolecules and cellular processes in living systems with high spatial and temporal resolution, through direct monitoring of the behaviors of fluorescent molecules. Fluorescent probes that emit a specific intensity or wavelength in response to the recognition of or reaction with a specific biomolecule are essential molecular tools for fluorescence imaging. To date, a large number of fluorescent probes have been designed using different strategies for various applications.[1–4] Fluorescent probes can be classified into two types, intensity-changing or ratiometric fluorescent probes. The major advantage of ratiometric fluorescent probes, which are based on the ratio of fluorescence emission or excitation intensities at two different wavelengths, is that they are less sensitive to errors associated with probe concentration, photo-bleaching, instrument sensitivity, and environmental effects.[5–8] However, ratiometric fluorescent probes having intrinsic fluorescence might obscure a change in the intracellular signal if background fluorescence is present outside the cell, because the synthetic probes must be loaded into the cells. Thus, extracellular background fluorescence must be minimized, especially for the intracellular application of these synthetic probes.[9] To this end, latent ratiometric fluorescent probes exhibiting a two-stage fluorescence response were considered.[10] These types of probes have no fluorescence (‘off-state’) prior to reacting with an external stimulus such as an intracellular enzyme; that is, the stimulus acts to ‘turn on’ the fluorescence. Thereafter, the activated probe can be used to detect the target analyte in a ratiometric manner. Through a sequence of responses, the latent ratiometric fluorescent probe can be activated and used to detect the target analyte inside the cell.

Based on the foregoing discussion, we recently reported a rational strategy for the design of a latent ratiometric fluorescent probe for monitoring intracellular pH (pH_i_), i.e. a latent ratiometric fluorescent pH_i_ probe,[10–13] since tracking the dynamics of pH_i_ is crucial to understanding the regulation mechanisms of several physiological functions of cells and tissues.[14,15] It is well known that seminaphthorhodafluor (SNARF-OH) [16] and its derivatives [17–19] are most commonly used as ratiometric fluorescent pH probes. In our previous study, SNARF-OR derivatives, in which the phenolic group was protected by hydrophobic substituents (indicated as R), formed colorless and non-fluorescent self-assembled clusters in the ‘off-state’ in aqueous solutions.[10–13] This process was named as self-assembly-induced lactone formation (SAILac).[10] The fluorescence of SNARF-OH as the ratiometric fluorescent pH probe could be initiated by converting the self-assembled cluster into disassembled SNARF-OH monomers (‘on-state’). Based on these findings, we designed latent fluorescent pH_i_ probes having no background extracellular fluorescence, which can be activated from the non-fluorescent self-assembled cluster state to the fluorescent monomeric state by endogenous enzymes such as nitroreductase [11] and esterase [12] in cell cultures. However, the requirement of such specific enzyme activation would not be universally achievable, because there are several cell lines that have low levels of intracellular enzyme activity such as esterase.[14] It should also be noted that temporal control of the fluorescence activation has not been realized yet because the reaction with the intracellular enzyme occurs continuously after cellular uptake.

In this paper, we report a fluorescent pH probe in which the off and on state of fluorescence are temporally controlled. To achieve this control, we focused on a photoremovable protecting group, also well known as a caging group, which can be removed by brief ultraviolet (UV) irradiation to release the molecules.[20,21] Our designed caged-SNARF derivative existed as a colorless and nonfluorescent self-assembled cluster in aqueous solution prior to UV irradiation. In contrast to our previous knowledge, the UV-irradiated uncaged-SNARF derivatives maintained the self-assembled cluster state, but the cluster showed strong pH-responsive fluorescence. This unique pH-responsive fluorescent cluster could be used as a dual-emissive ratiometric fluorescent pH probe in both test tube and cell cultures.

## 2. Results and discussion

**Figure 1.**
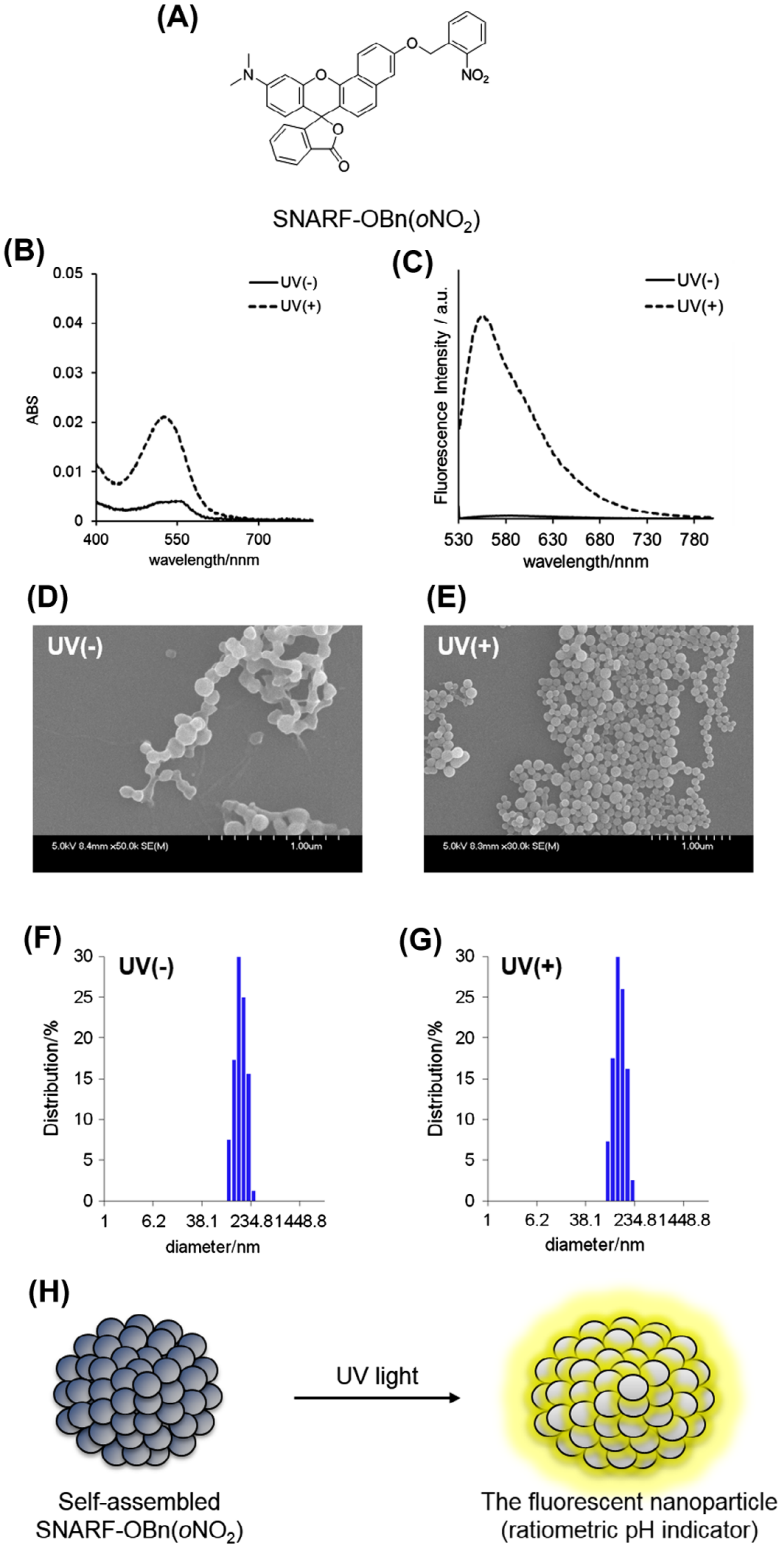
(A) The structure of SNARF-OBn(*o*NO_2_). (B) The absorption and (C) the emission spectra (excited at 500 nm) of SNARF-OBn(*o*NO_2_) before and after UV irradiation, where solid lines indicate SNARF-OBn(*o*NO_2_) before UV irradiation, and dashed lines indicate SNARF-OBn(*o*NO_2_) after UV irradiation. [SNARF derivatives] = 10 μM in 10 mM Tris, Hepes, and acetate buffer at pH7.0. (D, E) SEM image of SNARF-OBn(*o*NO_2_) (D) before and (E) after UV irradiation. (F, G) DLS analysis of SNARF-OBn(*o*NO_2_) (F) before and (G) after UV irradiation. (H) Schematic illustration of the photo-activated pH sensitive fluorescent cluster comprising self-assembled SNARF-OBn(*o*NO_2_).

The SNARF derivative, in which the phenolic group of SNARF-OH (Figure S1A) was protected by the photolabile *o*-nitrobenzyl group(SNARF-OBn(*o*NO_2_), Figure 1(A)), was designed and synthesized as shown in Scheme S1. Based on previous reports,[10–13] a self-assembled cluster comprising the SNARF scaffold could be rationally designed by tuning the calculated Hansch-Fujita hydrophobic parameter (π) of the introduced substituents. When π > 0, the SNARF derivatives might be shifted toward the self-assembled cluster state. The π value of the *o*-nitrobenzyl group was calculated to be 0.76; thus, SNARF-OBn(*o*NO_2_) could potentially form self-assembled clusters.

An evaluation of the spectroscopic properties of SNARF-OBn(*o*NO_2_) revealed that it produced absorption and fluorescence emission spectra distinct from those of SNARF-OH (Figure S1(B) and S1(C)). While SNARF-OH showed maximum absorption and fluorescence emission around 551 and 631 nm at pH 7.0, respectively, SNARF-OBn(*o*NO_2_) did not exhibit any significant absorption above 400 nm or fluorescence emission (Figure S1(B) and S1(C)). Our previous studies on the self-assembled cluster formation of SNARF derivatives suggested that the disappearance of absorption, as in the case of SNARF-OBn(*o*NO_2_), indicated self-assembled cluster formation based on the SAILac mechanism.[10] After UV irradiation, increments were observed in the absorption (λ_Abs_ = 525 nm) and fluorescence emission (λ_em_ = 555 nm) of SNARF-OBn(*o*NO_2_) at pH 7.0 (Figure 1(B) and 1(C)). The UV-irradiated SNARF-OBn(*o*NO_2_) showed approximately 450-fold higher fluorescence than prior to UV irradiation. According to the general characteristics of a photoremovable protecting group, the released compound (in this case, SNARF-OH) should exhibit the original characteristics of the monomer after photo-irradiation.[20]^1^ However, the characteristics of the absorption and fluorescence emission spectra of UV-irradiated SNARF-OBn(*o*NO_2_) were significantly different in comparison with SNARF-OH (λ_Abs_ = 551 nm, λ_em_ = 631 nm). We hypothesized that the characteristics of the self-assembled SNARF-OBn(*o*NO_2_) clusters caused the change in the photophysical properties. To clarify this hypothesis, morphological analyses of SNARF-OBn(*o*NO_2_) were performed both before and after UV irradiation, using scanning electron microscopy (SEM) and dynamic light scattering (DLS) (Figure 1(D–G)). SEM images of SNARF-OBn(*o*NO_2_) both before and after UV irradiation showed the formation of particles of approximately 100 nm in size (Figure 1(D) and 1(E)). DLS measurements of SNARF-OBn(*o*NO_2_) before and after UV irradiation showed particles with mean diameters of 147 ± 32 nm (Figure 1(F)) and 124 ± 27 nm (Figure 1(G)), respectively.^2^ These results suggested that SNARF-OBn(*o*NO_2_) exists as self-assembled clusters, both before and after UV irradiation. UV-irradiated SNARF-OBn(*o*NO_2_) maintained the self-assembled cluster state unlike our previously reported enzyme-responsive self-assembled SNARF derivatives, which disintegrated after enzyme treatment.[10] These results were strongly supported by size-exclusion chromatographic analyses of SNARF-OBn(*o*NO_2_) before and after UV irradiation (Figure S2). For SNARF-OBn(*o*NO_2_) both before and after UV irradiation, faster elution (eluted fraction from 3.5 to 4.5 ml) indicated the existence of the self-assembled clusters and little or no SNARF-OH monomer, which would have eluted more slowly (eluted fraction from 10 to 25 ml) (Figure S2(c)). These results indicated that UV-irradiated SNARF-OBn(*o*NO_2_) did not exist as diffusible SNARF-OH monomers but as self-assembled clusters that showed strong fluorescence (Figure 1(H)), unlike our previously reported self-assembled SNARF derivatives, although the reasons for the change in the photophysical properties of the UV-irradiated SNARF-OBn(*o*NO_2_) still remain ambiguous.^1^

**Figure 2.**
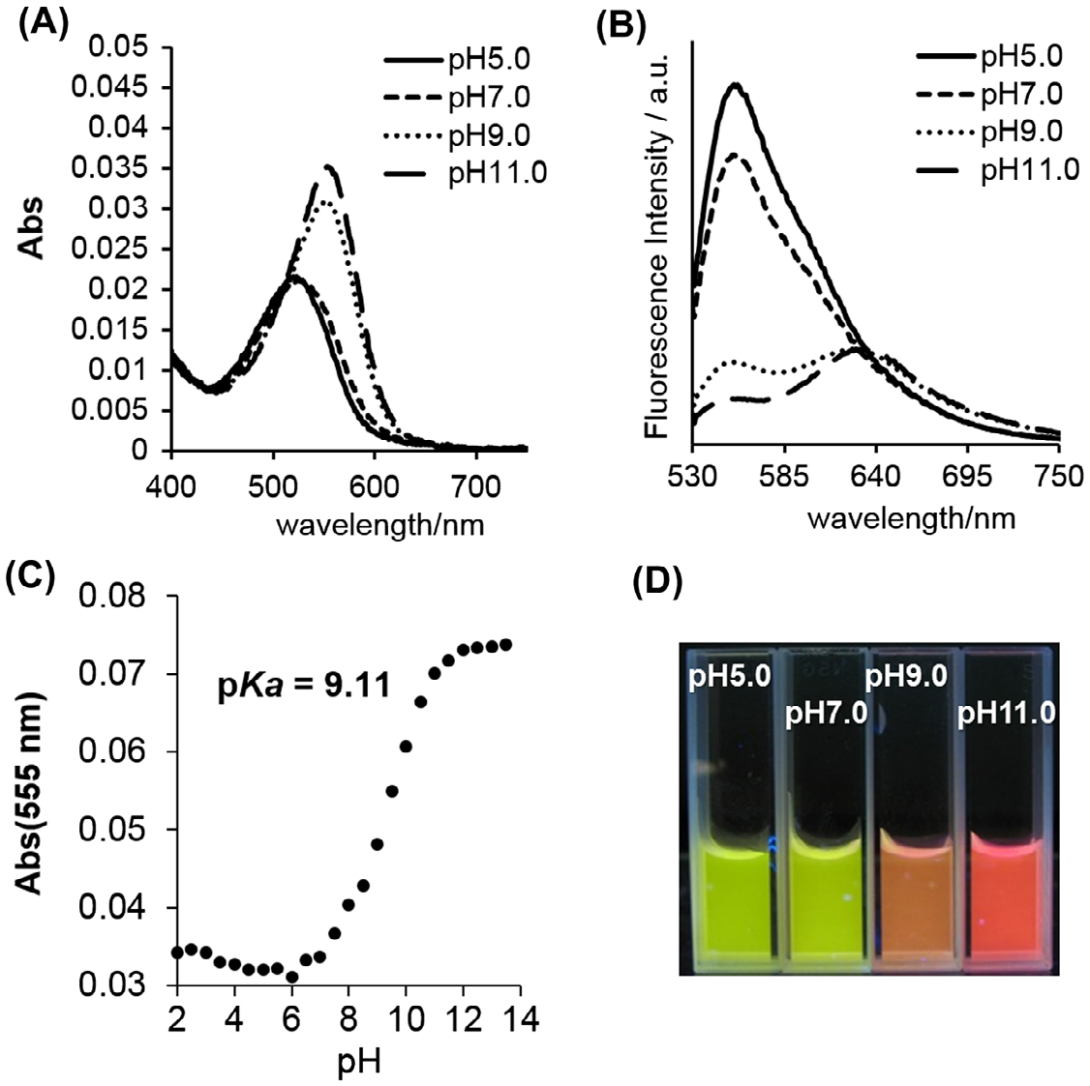
(A) The absorption spectra of UV-irradiated SNARF-OBn(*o*NO_2_) at different pH. (B) The emission spectra (excited at 500 nm) of UV-irradiated SNARF-OBn(*o*NO_2_) at different pH. (C) p*K*_a_ determination of UV-irradiated SNARF-OBn(*o*NO_2_) (see also Figure S3). (D) Photograph of UV-irradiated SNARF-OBn(*o*NO_2_) at different pH. [SNARF derivatives] = 10 μM in 10 mM Tris, Hepes, and acetate buffer (pH conditions for each spectrum were shown in these figures).

To investigate the unique characteristics of the fluorescent self-assembled cluster of UV-irradiated SNARF-OBn(*o*NO_2_), the pH sensitivity, which is a significant characteristic of SNARF-OH,[14–19] was evaluated (Table 1 and Figure 2). The fluorescent cluster showed pH-dependent spectral changes as shown in Figure 2. The maximum absorption and fluorescence emission at pH 5.0 or pH 12.0 were determined at 520 and 555 nm or at 553 and 631 nm, respectively. Both the fluorescence quantum yield (Φ) and the fluorescence lifetime (τ) of the clusters exhibited higher value at pH 5.0 than at pH 12.0. The p*K*_a_ of the fluorescent cluster was determined as 9.11 (Figures 2(c) and S3), and the reversibility of pH response of UV-irradiated SNARF-OBn(*o*NO_2_) was confirmed (Figure S4). As compared with SNARF-OH, the fluorescent cluster had different photophysical properties, though both comprised SNARF-OH and/or SNARF derivatives. These results indicated that the fluorescent cluster has unique characteristics as a dual-emissive ratiometric fluorescent pH probe (Figure 2(d)).

**Table 1.** Photophysical properties of UV-irradiated SNARF-OBn(*o*NO_2_) and SNARF-OH.

	λ_Abs_ (nm)^a^	λ_Abs_ (nm)^b^	λ_Em_ (nm)^a^	λ_Em_ (nm)^b^	Φ^a^	Φ^b^	τ (ns)^a^	τ (ns)^b^	p*K*_a_
UV-irradiated SNARF-OBn(*o*NO_2_) (fluorescent cluster)	520	553	555	553, 631	0.21	0.01	4.34	0.53, 1.88	9.11
SNARF-OH	523, 546	574	585	638	0.04	0.11	0.43	1.58	7.62^c^

^a^ At pH5.0;

^b^ at pH12.0;

^c^ from the previous report.[19]

**Figure 3.**
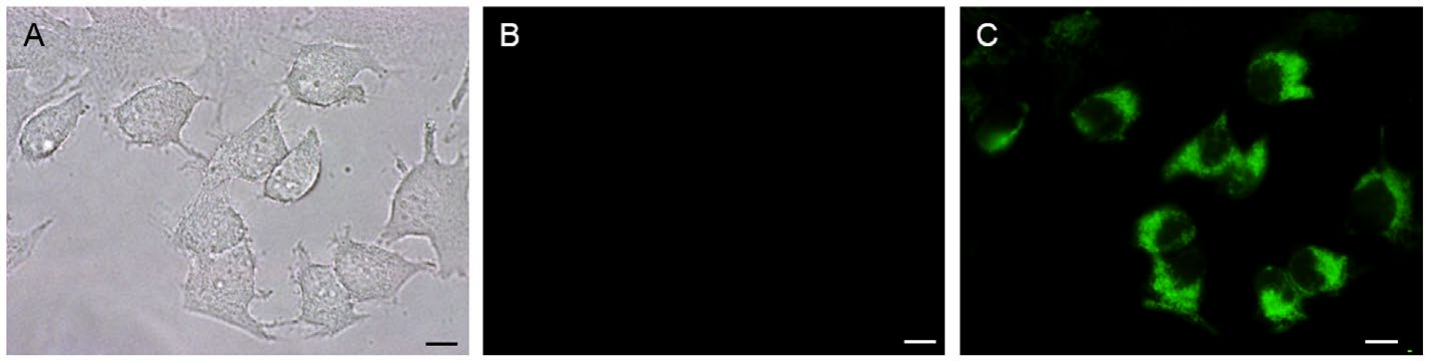
(A) Bright-field transmission image; (B, C) fluorescence images of HeLa cells pre-treated with SNARF-OBn(*o*NO_2_) (B) before and (C) after UV-irradiation for 15 min (scale bars, 10 μm).

Next, the properties of SNARF-OBn(*o*NO_2_) as a photo-activatable pH-responsive fluorescent cluster were evaluated for suitability as a pH_i_ probe. HeLa cells were incubated with non-UV-irradiated SNARF-OBn(*o*NO_2_) for 1 h, and the fluorescence microscopy analysis was performed after exchanging the medium (Figure 3). As shown in Figure 3(B), no fluorescence signals from SNARF-OBn(*o*NO_2_) were observed before UV irradiation. In contrast, after UV irradiation for 15 min, strong fluorescence from SNARF-OBn(*o*NO_2_) was observed only inside the HeLa cells as punctuate foci (Figures 3(C) and S7(B)). Figure S6 shows the changes in the fluorescence spectra of SNARF-OBn(*o*NO_2_) before and after UV irradiation inside the cells, measured using a microplate reader. The significant increment of fluorescence was only observed after UV irradiation, similar to the observation in fluorescence microscopy analysis. The maximum fluorescence emission was observed at 555 nm as with the observation in test tube. These results suggested that the self-assembled SNARF-OBn(*o*NO_2_) performed in the HeLa cell culture as well as in the test tube. To clarify the mechanism of the cellular uptake of the self-assembled SNARF-OBn(*o*NO_2_), the uptake efficiencies of SNARF-OBn(*o*NO_2_) at different temperatures were compared. HeLa cells were incubated with non-UV-irradiated SNARF-OBn(*o*NO_2_) for 1 h at 4 °C or 37 °C, and the media were exchanged. Then, the fluorescence intensity increments between before and after UV irradiation were measured by a microplate reader. No significant fluorescence increment was observed after incubation at 4 °C, in contrast to that at 37 °C (Figure S7(E)). The same results were also confirmed by microscopic analysis (Figure S7(A–D)). Since endocytosis is an energy-dependent pathway that is suppressed at low temperatures,[22,23] these results suggested that the internalization of the self-assembled SNARF-OBn(*o*NO_2_) involved a kind of endocytosis similar to the previously reported self-assembled SNARF derivatives.

Finally, the change in intracellular pH was monitored by intracellular UV-irradiated SNARF-OBn(*o*NO_2_). HeLa cells incorporating SNARF-OBn(*o*NO_2_) were prepared and irradiated with UV light by the same process. The medium was exchanged for buffer having different pH values (pH 6.0–9.0) with nigericin, which is an ionophore to equilibrate pH_i_ and extracellular pH. The pH-dependent spectral changes are shown in Figure S8. These results strongly indicated that UV-irradiated SNARF-OBn(*o*NO_2_) has potential as a dual-emissive ratiometric fluorescent pH_i_ probe.

## 3. Conclusion

We developed SNARF-OBn(*o*NO_2_) as a new photo-activatable fluorescent cluster that could be used as a dual-emissive ratiometric fluorescent pH_i_ probe. SNARF-OBn(*o*NO_2_) formed a colorless and nonfluorescent self-assembled cluster in aqueous solution. After UV irradiation, the self-assembled cluster state was retained and showed a large fluorescence enhancement with unique fluorescence properties, including pH responsivity. These properties enabled the use of SNARF-OBn(*o*NO_2_) as a dual-emissive ratiometric fluorescent pH_i_ probe. Though the fluorescent cluster comprising the SNARF scaffold has a basic p*K*_a_ and, thus, could not be used to measure near-neutral and acidic pH_i_ changes, the p*K*_a_ value of the fluorescent cluster might be fine-tuned using low p*K*_a_ SNARF derivatives, as reported previously.[17,19] We believe that the pH-responsive fluorescent clusters activated by UV irradiation have great potential for future application in monitoring pH_i_ changes in cells or tissues.

## Disclosure statement

No potential conflict of interest was reported by the authors.

## Funding

This work was supported in part by a Grant-in-Aid for Scientific Research from the Ministry of Education, Culture, Sports, Science and Technology, Japan to EN [number 21710232, 24107513, 26107710].

## Supplementary material

The supplementary material for this paper is available online at http://dx.doi.org/10.1080/14686996.2016.1204888

## Supplementary Material

Supporting_information_rerevised_20160616.pdf

## Footnotes

^1^ To check the products after UV irradiation, HPLC profiles of SNARF-OBn(*o*NO_2_) were obtained before or after UV irradiation (Figure S5). The main product was SNARF-OH, and two other peaks were observed, which may correspond to a reaction intermediate or a byproduct.

^2^ The size of the self-assembled clusters at different pH values was also measured by DLS as shown in Table S1. The formation of the self-assembled clusters was confirmed in a wide pH range (from pH 4.0 to pH 10.0), in agreement with our previous report.[10]

## References

[CIT0001] Lavis L, Rains RT (2008). Bright ideas for chemical biology. ACS Chem. Biol.

[CIT0002] Kobayashi M, Ogawa R, Alford P (2010). New strategies for fluorescent probe design in medical diagnostic imaging. Chem. Rev.

[CIT0003] Chan J, Dodani SC, Chang CJ (2012). Reaction-based small-molecule fluorescent probes for chemoselective bioimaging. Nat. Chem.

[CIT0004] Demchenko AP (2005). Optimization of fluorescence response in the design of molecular biosensors. Anal. Biochem.

[CIT0005] Johnsson N, Johnsson K (2007). Chemical tools for biomolecular imaging. ACS Chem. Biol.

[CIT0006] Doussineau T, Schultz A, Lapresta-Fernandez A (2010). On the design of fluorescent ratiometric nanosensors. Chem.–Eur. J.

[CIT0007] Demchenko AP (2010). The concept of λ-ratiometry in fluorescence sensing and imaging. J. Fluoresc.

[CIT0008] Yuan L, Lin W, Zheng L (2013). FRET-based small-molecule fluorescent probes: rational design and bioimaging applications. Acc. Chem. Res.

[CIT0009] Dustin LB (2000). Ratiometric analysis of calcium mobilization. Clin. Appl. Immunol. Rev.

[CIT0010] Nakata E, Yukimachi Y, Nazumi Y (2014). A novel strategy to design latent ratiometric fluorescent pH probes based on self-assembled SNARF derivatives. RSC Adv.

[CIT0011] Nakata E, Yukimachi Y, Kariyazono H (2009). Design of a bioreductively-activated fluorescent pH probe for tumor hypoxia imaging. Bioorg. Med. Chem.

[CIT0012] Nakata E, Yukimachi Y, Nazumi Y (2010). A newly designed cell-permeable SNARF derivative as an effective intracellular pH indicator. Chem. Commun.

[CIT0013] Nakata E, Nazumi Y, Yukimachi Y (2015). Self-assembled fluorescent nanoprobe for the detection of fluoride ions in aqueous solutions. Bull. Chem. Soc. Jpn.

[CIT0014] Haugland RP, Spence MTZ, Johnson ID (2005). The handbook: a guide to fluorescent probes and labeling technologies. Molecular Probes Eugene OR.

[CIT0015] Han J, Burgess K (2010). Fluorescent indicators for intracellular pH. Chem. Rev.

[CIT0016] Whitaker JE, Haugland RP, Prendergast FG (1991). Spectral and photophysical studies of benzo[c]xanthene dyes: dual emission pH sensors. Anal. Biochem.

[CIT0017] Liu J, Diwu Z, Leung WY (2001). Synthesis and photophysical properties of new fluorinated benzo[c]xanthene dyes as intracellular pH indicators. Bioorg. Med. Chem. Lett.

[CIT0018] Nakata E, Yukimachi Y, Nazumi Y (2010). Design of a SNARF-based ratiometric fluorescent probe for esterase. Chem. Lett.

[CIT0019] Nakata E, Nazumi Y, Yukimachi Y (2011). Synthesis and photophysical properties of new SNARF derivatives as dual emission pH sensors. Bioorg. Med. Chem. Lett.

[CIT0020] Klán P, Šolomek T, Bochet CG (2013). Photoremovable protecting groups in chemistry and biology: reaction mechanisms and efficacy. Chem. Rev.

[CIT0021] Kobayashi T, Urano Y, Kamiya M (2007). Highly Activatable and rapidly releasable caged fluorescein Derivatives. J. Am. Chem. Soc.

[CIT0022] Ivanov AI (2008). Pharmacological inhibition of endocytic pathways: is it specific enough to be useful?. Methods Mol. Biol.

[CIT0023] Nakase I, Takeuchi T, Tanaka G (2008). Methodological and cellular aspects that govern the internalization mechanisms of arginine-rich cell-penetrating peptides. Adv. Drug Delivery Rev.

