# Supplementary material for: Latent pH-responsive ratiometric fluorescent cluster based on self-assembled photoactivated SNARF derivatives
Source: Sci Technol Adv Mater. 2016 Aug 2;17(1):431–6. doi: 10.1080/14686996.2016.1204888 (PMC5101900; doi:10.1080/14686996.2016.1204888)
Supplement: Supporting_information_rerevised_20160616.pdf [file tsta_a_1204888_sm5329.pdf]

Supporting Information

Latent pH responsive ratiometric fluorescent cluster based on self-assembled photoactivated SNARF derivative

Eiji Nakata[a*], Yoshihiro Yukimachi[b], Yoshihiro Uto[b], Hitoshi Hori[b], Takashi Morii[a]

[a]*Institute of Advanced Energy, Kyoto University, Uji, Kyoto 611-0011, Japan*

[b]Department of Life System, Institute of Technology and Science, Graduate School, The University of Tokushima, Minamijosanjimacho 2, Tokushima 770-8506, Japan

Material and Methods

1. General Procedures

^{1}H NMR spectra were recorded on a JEOL JNM-EX400 spectrometer (400 MHz) using tetramethylsilane as the internal standard. Chemical shifts are reported in ppm. Coupling constants are reported in Hz. IR spectra was measured in KBr pellets with a PerkinElmer 1600 spectrometer. HRMS was measured on a JEOL JMS-700 mass spectrometer using the FAB method. Elemental analyses were performed with a Yanako CHN recorder MT-5. Reactions were monitored by analytical TLC using Merk Silica Gel 60 F_{254} aluminum plates. Column chromatography was performed on Kanto Chemical Silica Gel 60 N (230–400 mesh). Absorption spectra and fluorescence spectra were recorded on a TECAN infinit M200 microplate reader. Dynamic light scattering (DLS) was measured with a DLS-7000 Photal Dynamic Laser Scattering Spectrometer from Otsuka Electronics. Scanning electron microscope (SEM) microscopy was measured with S-4700 Field Emission Scanning Electron Microscope from HITACHI. Size exclusion chromatography was performed with Sephadex™ G-25 M PD-10 Column from GE Healthcare. UV-irradiation in test tube was irradiated with TFX-20M UV Transilluminator fom Vilber Lourmat at 312 nm. UV-irradiation for cell cluture was irradiated with IX71N-22FL/PH (WU filter) from Olympus. Fluorescence microscopy was measured with IX71N-22FL/PH system (WIB filter) from Olympus. Fluorescence quantum yield (Φ) was measured by C9920-02 from Hamamatsu Photonics. Fluorescence lifetime (τ) was measured by Quantaurus-Tau from Hamamatsu Photonics. HPLC were performed on the μRPC C2/C18 SC column (2.1 × 100 mm, Pharmacia) with a Pharmacia Biotech SMART system. All chemicals were purchased from Wako Pure Chemical Industries, Ltd (Osaka, Japan), Kanto Chemical Co., Inc. (Tokyo, Japan), Tokyo Chemical Industry Co., Ltd (Tokyo, Japan), or Sigma–Aldrich Japan (Tokyo, Japan).

2. Synthesis

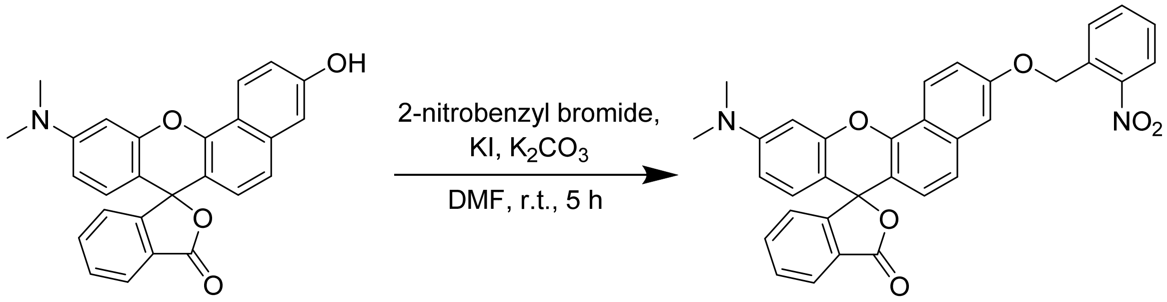

Scheme S1. Synthesis of SNARF-OBn(*o*NO_2)

2.1. Synthesis of 10-(Dimethylamino)-3-hydroxy-Spiro[7H-benzo[c]xanthene-7,1'(3'H)-isobenzofuran]-3'-one (seminaphthorhodafluor, SNARF-OH)

This was synthesized by previously-reported method as a violet solid.[S1]

2.2. Synthesis of 10-(Dimethylamino)-3-[(2-nitrophenyl)methoxy]-spiro-[7H-benzo[c]xanthene-7,1'(3'H)-isobenzofuran]-3'-one [SNARF-OBn(*o*NO_2)]

2-nitrobenzyl bromide (24.5 mg, 113.9 μmol) and KI (15.0 mg, 90.4 μmol) were stirred in 1 mL DMF at r.t. for 30 min, followed by addition to SNARF-OH (21.5 mg, 52.6 μmol) and K_2CO_3 (20.0 mg, 144.7 μmol) in 1.5 mL DMF. The mixture was stirred at r.t. for 5 h. The reaction mixture was dissolved in CH_2Cl_2 and washed with saturated aq. $NaHCO_3$ solution. The organic layer was dried over anhydrous Na_2SO_4, filtered, and evaporated. The residue was purified by silica gel column chromatography with CH_2Cl_2/AcOEt (1:0, 10:1, 5:1, stepwise) as the eluent to obtain the product as a pink solid (20.7 mg, 72%). ^{1}H NMR (Chloroform-*d*, 400 MHz): δ 8.53 (d, *J* = 9.3 Hz, 1H), 8.21 (dd, *J* = 1.2, 8.3 Hz, 1H), 8.05 (dd, *J* = 1.7, 6.3 Hz, 1H), 7.96 (dd, *J* = 0.7, 7.6 Hz, 1H), 7.71 (dt, *J* = 1.2, 7.7 Hz, 1H), 7.66-7.59 (m, 2H), 7.52 (dt, *J* = 0.7, 8.0 Hz, 1H), 7.90 (dd, *J* = 2.6, 9.1 Hz, 1H), 7.34-7.14 (m, 4H), 6.74 (d, 1H), 6.76-6.73 (m, 1H), 6.48 (dd, *J* = 2.6, 8.9 Hz, 1H), 5.64 (s, 2H), 3.04 (s, 6H). FT-IR (KBr; cm^{-1}): 2919.6, 1748.8, 1627.4, 1557.8, 1526.1, 1471.5, 1417.6, 1360.6, 1338.7, 1284.3, 1248.3, 1110.8, 1037.6, 862.6, 848.0, 826.4, 791.7, 759.9, 724.1, 694.7, 662.1, 613.5, 524.2. HR-MS (FAB^+): m/z calcd for $[M + H]^+$, 545.1707; found, 545.1736. Anal. Calcd for SNARF-OBn(*o*NO_2) + $2H_2O$ ($C_{33}H_{28}N_2O_8$): C, 68.27; H, 4.86; N, 4.83. Found: C, 68.32; H, 4.72; N, 5.16.

3. Analytical methods

3.1. Absorption spectrophotometry and Fluorescence spectrophotometry

Absorption spectra and Fluorescence spectra (with excitation at 500 nm) were measured in 10 mM Tris, Hepes and Acetate buffer (various pH range as shown in each figure) at 25 ± 1 °C.

3-2. Measurement of fluorescence quantum yield

The absorbance at 555 nm of UV irradiated SNARF-OBn(*o*NO_2) (20 μM) was measured and the pK_a value was determined by eq 1.

$$pK_a = pH + \log\{(A-Aa)/(Ab-A)\} \quad (1)$$

A; ABS at each pH, Aa; ABS at acidic pH, Ab; ABS at basic pH

3-3. Measurement of fluorescence quantum yield

The fluorescence quantum yield (Φ) (λ_{ex} = 405 nm) was determined by using eq 2.

$$\Phi = \text{Radiated Photon / Absorbed photon} \quad (2)$$

3-4. Measurement of fluorescence lifetime

The fluorescence lifetime was measured at 405 nm excitation and fluorescence signal was collected by C11367-01 detector.

3-5. DLS analysis

DLS measurements were taken by placing SNARF-OBn(*o*NO_2) (10 μM) in 10 mM Tris, Hepes and acetate buffer with or without UV irradiation for 30 min.

3-6. SEM imaging

SNARF-OBn(*o*NO_2) (10 μM) with or without UV irradiation (for 30 min) in distilled water were dropped on a glass plate (18 mm × 18 mm) and dried at room temperature for 24 h. Pt-Pd coating was applied for 60 s.

3-7. Size-exclusion chromatographic analysis

SNARF-OH or SNARF-OBn(*o*NO_2) (10 μM) with or without UV irradiation for 30 min in 10 mM Tris, HEPES, and acetate buffer at pH 7.0, were loaded onto the pre-packed gel filtration column (PD-10, GE Healthcare) and eluted with 10 mM Tris, HEPES, and acetate buffer at pH 7.0. The eluted fraction was evaluated with UV-vis spectra by Infnit M200 (Tecan).

3-8. HPLC analysis

SNARF-OBn(*o*NO_2) (10 μM) with or without UV irradiation for 30 min in 10 mM Tris, HEPES, and acetate buffer at pH 7.0, were analyzed by HPLC. The HPLC solvents were water containing 0.1% trifluoroacetic acid

(TFA) (solvent A) and 95% acetonitrile and 5% water containing 0.1% TFA (solvent B). HPLC condition : solvent A : solvent B = 100 : 0 (0 min) – 100 : 0 (5 min) – 0 : 100 (30 min), flow rate 200 μl/min, detection by UV (534 nm).

3-9. Live cell imaging for HeLa cells

HeLa cells were cultured in Eagle minimum essential medium without phenol red containing 12.5 % (v/v) fetal bovine serum and kanamycin (60 mg/L) (written for EMEM (+)) at 37 °C in 5% CO_2.For live cell imaging by fluorescence microscopy, HeLa cells (5 × 10^4 cells) in the late log phase were seeded in a 35-mm glass base dish. After growth in a CO_2 incubator for 24 h, the culture medium was exchanged to 1 mL of flesh EMEM (+). Then, 1 mL of EMEM (+) containing SNARF-OBn(*o*NO_2) (20 μM) was added (final concentration of SNARF-OBn(*o*NO_2) was 10 μM). After 1 h incubation, the cells were washed with EMEM (+) and observed with fluorescence microscopy though WIB filter. And then, UV light were irradiated to HeLa cells for 15 min through WU filter and observed with fluorescence microscopy though WIB filter under the same condition. In order to check the temperature dependency, the cells pre-incubated for 1 h in EMEM (+) at 4 or 37 ˚C were used.

3-10. Measurement of intracellular fluorescence spectra

For measurement of intracellular fluorescence spectra, the suspension of HeLa cells (1.0 × 10^6 cells/mL) in EMEM (+) was treated with 10 μM SNARF-OBn(*o*NO_2) and incubated at 4 or 37°C for 1h. The suspension was centrifuged at 1000 rpm for 5 min, diluted by EMEM (+), and transferred to a 96 well microplate (FIA black plate from greiner bio-one), and then subjected to fluorescence analysis using Infinit M200 (Tecan). In the case of intracellular pH measurement, The suspension was diluted by assay buffer (140 mM KOH, 2 mM $CaCl_2$, 1 mM $MgCl_2$, 5 mM D(+)-glucose, 20 mM Tris and MES) having different pH values (pH 6.0-9.0) with nigericin (final concentration of 10 μg/mL).

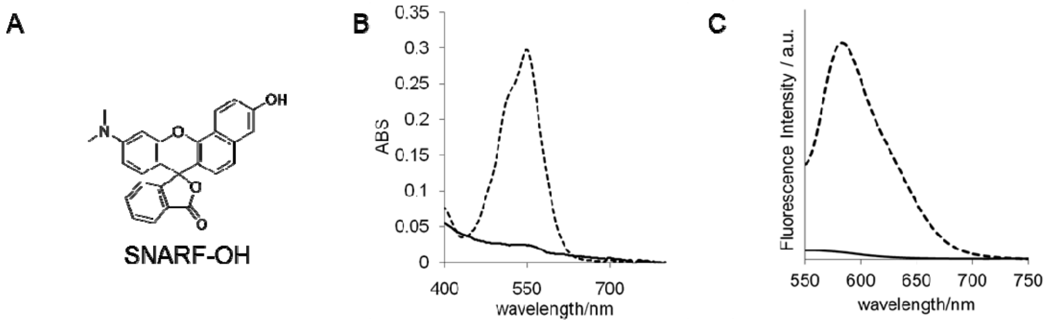

Fig. S1 (A) The structure of SNARF-OH. (B) The absorption spectra and (C) the emission spectra (excited at 488 nm) of SNARF-OBn(*o*NO_2) and SNARF-OH. [(B and C) solid line : SNARF-OBn(*o*NO_2), dashed line :

SNARF-OH]. [SNARF derivatives] = 10 μM in pH7.0 10 mM Tris, Hepes, and acetate buffer.

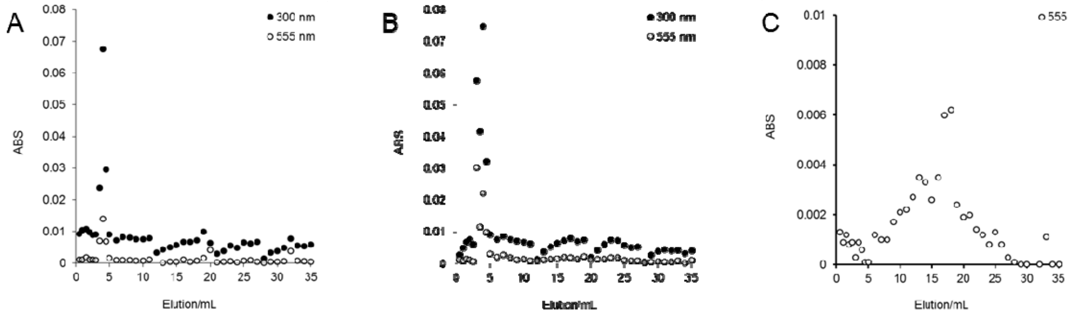

Figure S2. The size exclusion gel chromatographic analysis of SNARF-OBn(oNO$_2$) before and after UV irradiation and SNARF-OH. Fraction curves of Absorbance (ABS) of SNARF-OBn(oNO$_2$) (A) before and (B) after UV irradiation and (C) SNARF-OH, respectively. Filled circle: ABS at 300 nm; opened circle: ABS at 555 nm

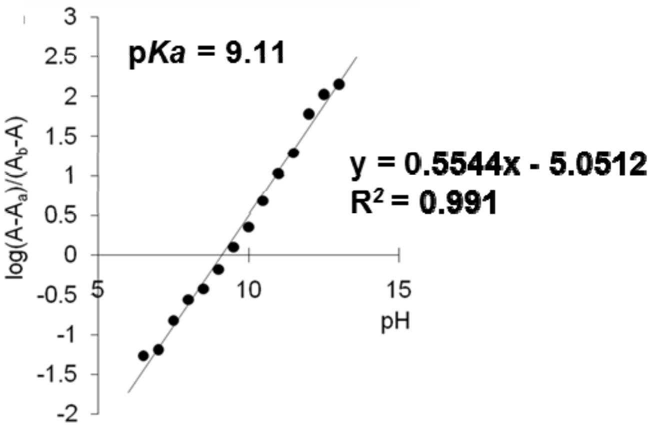

Fig. S3 Determination of pK_a from titration curve at 555 nm.

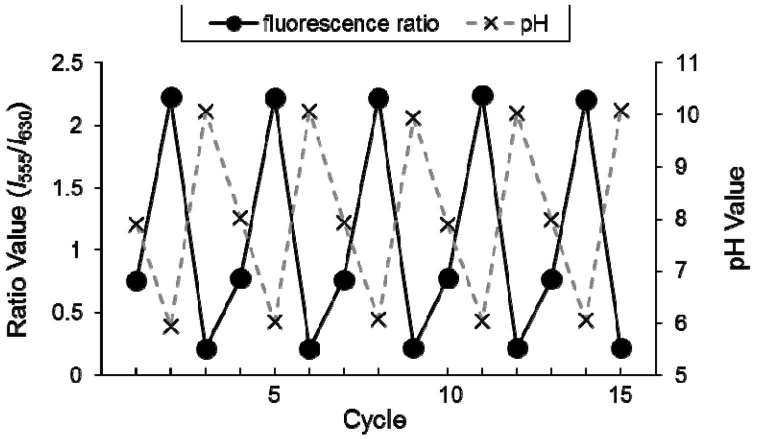

Fig. S4 The reversible pH responsibility of UV-irradiated SNARF-OBn(*o*NO_2).

Table S1 DLS data of UV-irradiated SNARF-OBn(*o*NO_2) under various pH conditions

Solutions [a]	Concentration	Diameter (nm)
pH 4.0	10 μM	373.5 ± 82.3
pH 6.0	10 μM	185.7 ± 42.2
pH 7.0	10 μM	123.6 ± 27.4
pH 8.0	10 μM	107.2 ± 24.2
pH 10.0	10 μM	35.6 ± 6.8

[a] 10 mM Tris, HEPES, and Acetate buffer

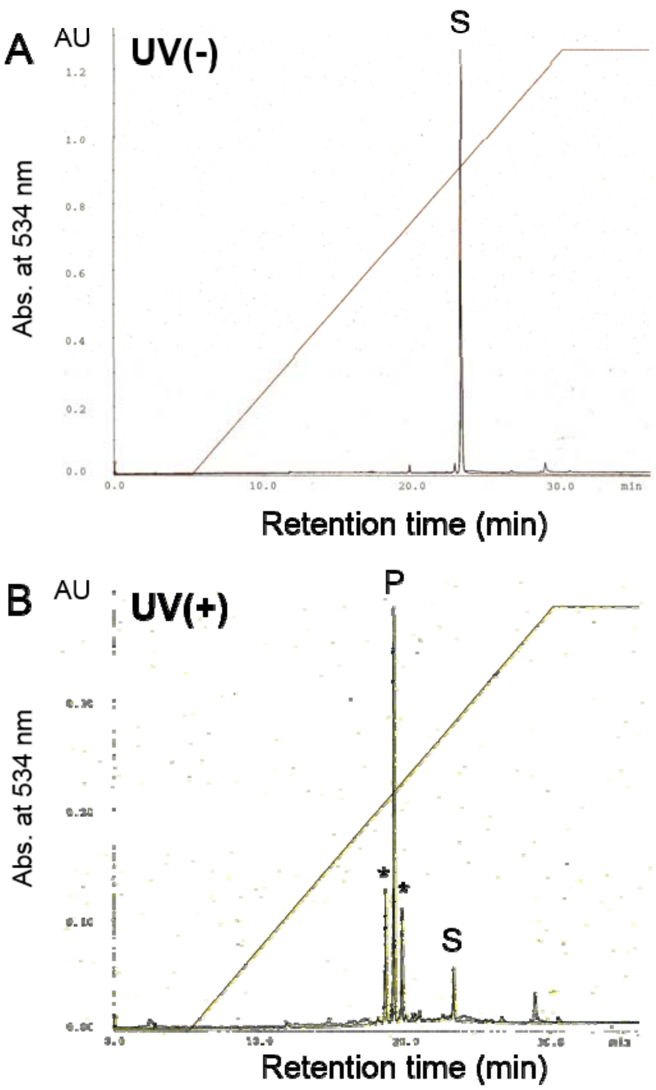

Fig. S5 HPLC profiles (detected by UV at 534 nm) of SNARF-OBn($o$$NO_2$) (A) before or (B) after UV irradiation. S : SNARF-OBn($o$$NO_2$), P : SNARF-OH. Though the peaks indicated as symbol '*' could not be identified, they possibly corresponds to a reaction intermediate or byproduct.

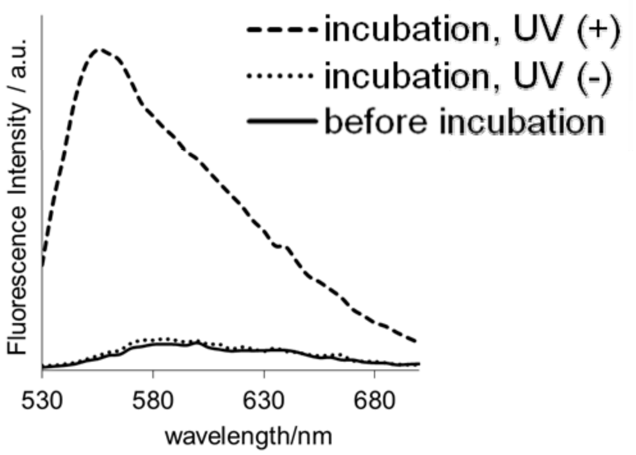

Fig. S6 Intracellular fluorescence spectra (excited at 500 nm) of SNARF-OBn(*o*NO_2) in HeLa cells, measured by microplate reader before (dot line) and after (dashed line) UV irradiation.

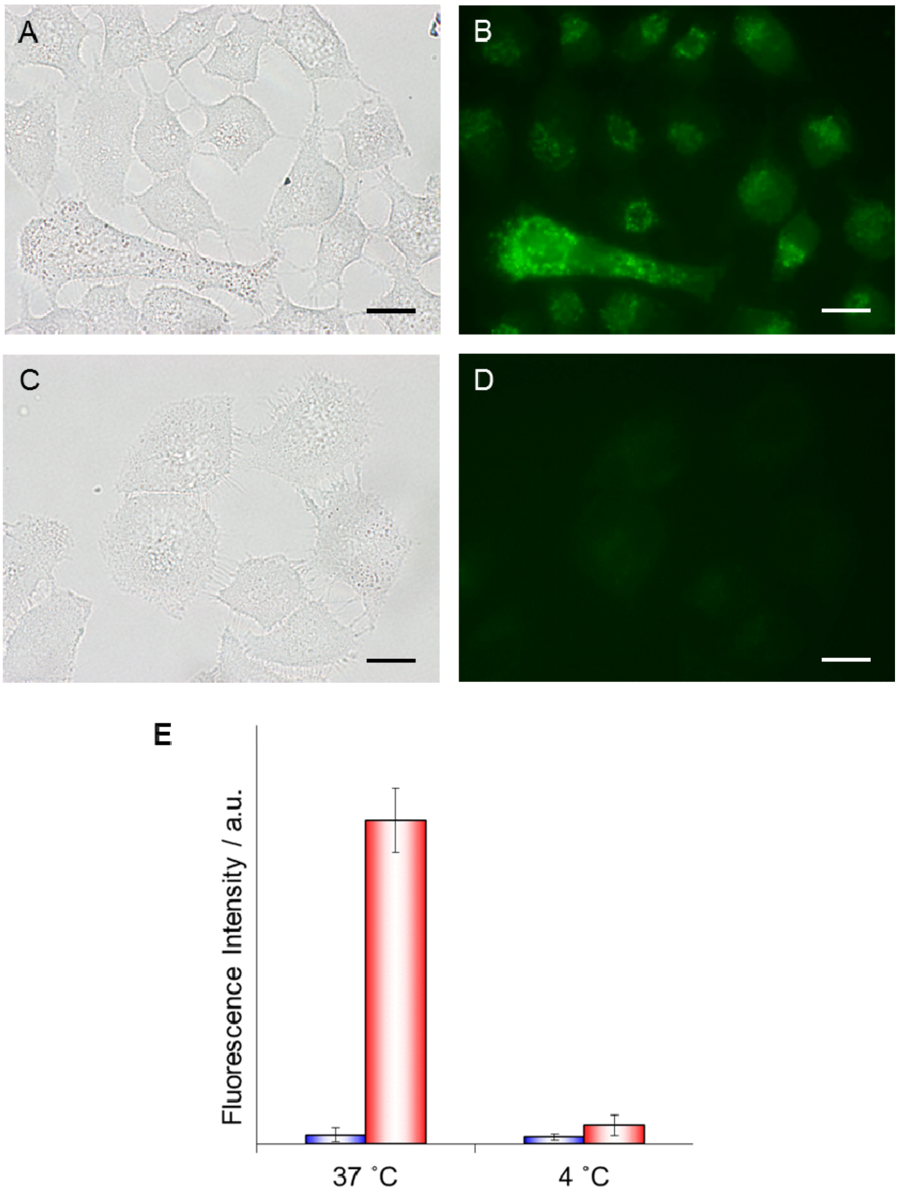

Fig. S7 (A, C) Bright-field transmission and (B, D) fluorescence imaging of SNARF-OBn(*o*NO_2) treated HeLa cells after UV irradiation for 15 min (Scale bars, 10 μm). The cells were pre-incubated with non-UV-irradiated SNARF-OBn(*o*NO_2) at (A, B) 37 ˚C or (C, D) 4 ˚C, respectively. (E) Comparison of fluorescence intensity at 631 nm before (blue bar) and after (red bar) UV irradiation. Error bars were generated as the standard deviation of the mean from three replicates.

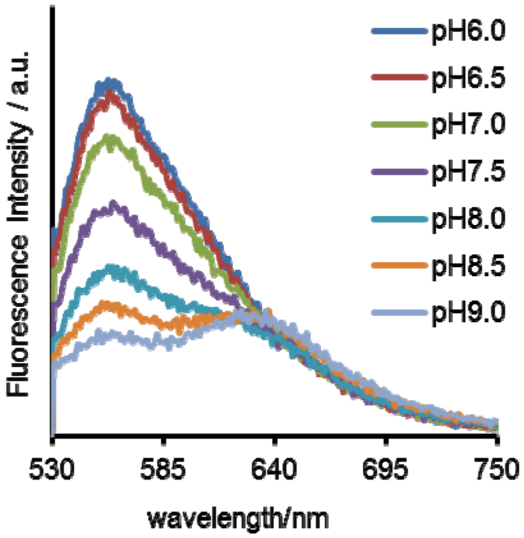

Fig. S8 Intracellular fluorescence spectra (excited at 500 nm) of UV-irradiated SNARF-OBn(*o*NO_2) in HeLa cells, measured by microplate reader after addition of nigericin (10 mg/mL) in an assay buffer at various pH values (pH condition of each spectrum are shown in the figure).

Reference

S1 Haugland, R. P, and J. Whitaker. July 1990. Xanthene dyes having a fused (C) benzo ring. U.S. patent 4,945,171.
